# Characterization of Essential Oils Obtained from Abruzzo Autochthonous Plants: Antioxidant and Antimicrobial Activities Assessment for Food Application

**DOI:** 10.3390/foods7020019

**Published:** 2018-02-02

**Authors:** Marika Pellegrini, Antonella Ricci, Annalisa Serio, Clemencia Chaves-López, Giovanni Mazzarrino, Serena D’Amato, Claudio Lo Sterzo, Antonello Paparella

**Affiliations:** Facoltà di Bioscienze e Tecnologie Agro-Alimentari e Ambientali, Università degli Studi di Teramo, Via R. Balzarini 1, 64100 Teramo, Italy; mpellegrini@unite.it (M.P.); aserio@unite.it (A.S.); cchaveslopez@unite.it (C.C.-L.); g.mazzarrino@virgilio.it (G.M.); sdamato@unite.it (S.D.); closterzo@unite.it (C.L.S.); apaparella@unite.it (A.P.)

**Keywords:** essential oils, antimicrobial, antioxidant, GC-MS

## Abstract

In the present study, the essential oils (EOs) of some officinal plants from Abruzzo territory (Italy) were evaluated for their antimicrobial and antioxidant activities and their volatile fraction chemical characterization. The EOs were extracted from *Rosmarinus officinalis*, *Origanum vulgare*, *Salvia officinalis*, *Mentha piperita*, *Allium sativum*, *Foeniculum vulgare*, *Satureja montana*, *Thymus vulgaris* and *Coriandrum sativum* seeds. The antimicrobial activity was screened against thirteen Gram-positive and Gram-negative strains to determine the Minimal Inhibitory Concentration (MIC). The total phenolic content (TPC) and the antioxidant capacity (AOC) were assessed by means of Folin-Ciocâlteu method, and Trolox Equivalent Antioxidant Capacity with 2,2′-azinobis-(3-ethylbenzothiazoline-6-sulfonic acid (TEAC/ABTS), Ferric Reducing Antioxidant Power (FRAP) and 2,2-diphenyl-1-picrylhydrazyl (DPPH) assays respectively. Among the nine EOs tested, *T. vulgaris*, *S. montana*, *O. vulgare* and *C. sativum* EOs showed MIC values ranging from 0.625 to 5 μL/mL. The AOC and TPC results for these species were also interesting. The major components for these EOs were thymol for *T. vulgaris* (44%) and *O. vulgare* (40%), linalool (77%) for *C. sativum*, and carvacrol for *S. montana* (54%). The results allowed the study to establish that these EOs are good candidates for potential application as biopreservatives in foods and/or food manufacture environments.

## 1. Introduction

Food spoilage can be defined as the alteration of a product due to microbial, chemical, or physical mechanisms that lead a food to become undesirable or unacceptable for human consumption [1]. In food products manufacture, many effective preservation strategies are applied against food spoilage, involving mainly the employment of synthetic preservatives. However, the increasing negative consumer perception of synthetic additives and the worldwide growing problem of allergies, is causing the food industry to search for more effective preservation strategies [2].

An alternative strategy to synthetic chemical preservatives is represented by the employment of essential oils (EOs). Commonly employed in foods as aromatizing and flavoring agents [2], these plant volatile fractions can be exploited by the food industry for their antimicrobial [3,4] and antioxidant [5] properties. EOs, in fact, possess the ability to permeabilize the membrane of microorganisms, with consequent loss of vital intracellular constituents and interruption of the cellular metabolism and enzyme kinetics [6]. In addition, terpenes and terpenoids, alkaloids and phenolic compounds present in these volatile fractions are recognized antioxidant substances [7,8,9].

According to European Pharmacopeia, the EOs can be obtained by steam distillation or by hydrodistillation [10] and their yield and composition are influenced by the presence of several factors, such as location, climate, plant species, methodology and experimental procedures [11,12].

In the Italian territory, Abruzzo is one of the central area regions characterized by a multitude of environments and microclimates and with the richest flora of Italy and the Mediterranean basin [13]. In the Abruzzo territory, different plant species are cultivated and exploited for their therapeutic and alimentary properties [14]; among them, *Rosmarinus officinalis*, *Origanum vulgare*, *Salvia officinalis*, *Mentha piperita*, *Allium sativum*, *Foeniculum vulgare*, *Satureja montana*, *Thymus vulgaris*, and *Coriandrum sativum*, are the aromatic plants commonly employed in the Mediterranean diet [15,16,17].

In scientific literature, different data that showed the antimicrobial and antioxidant potentials of the EOs recovered from these vegetal species are reported [18,19,20,21]. However, limited data are available for the EOs obtained from these Abruzzo plant species. In this perspective, the aims of the study were the extraction and the Gas Chromatography-Mass Spectrometry (GC-MS) characterization of the EOs from Abruzzo officinal plants and assessment of their antimicrobial properties against several food-borne strains, as well as their antioxidant capacities and total phenolic contents.

## 2. Materials and Methods

### 2.1. Plant Material

The matrices were obtained from Abruzzo territory farmers. The cultures were obtained with organic agriculture and after the harvesting, the matrices were dried on fields and stored at room temperature in dry and dark conditions for few days. The matrices were then transferred in the laboratory for extractions and analyses. Regarding *A. sativum*, after the harvesting, the bulbs were transferred in the laboratory, cleaned and the resultant cloves were vacuum-packed and refrigerated until extraction and analyses.

### 2.2. Essential Oils Extraction

Essential oils were extracted from the matrices by means of an E0105 12 lt PLUS Essential Oils Extractor (Albrigi Luigi Srl, Verona, Italy). For all plant materials, after two hours of distillation, no significant volume increase was observed in the collector tube, thus all matrices were subjected to 2 h steam distillation, except for garlic cloves which were subjected to 2 h hydrodistillation. After extraction, the EOs were transferred to an amber glass vial with anhydrous sodium sulfate (Sigma Aldrich, Saint Louis, MO, USA), conditioned with argon and sealed. Each matrix extraction was conducted in triplicate. The collected EOs were stored under refrigeration at 4 °C.

### 2.3. Chemical Compositions of EOs

The GC-MS analyses of the EOs were carried out by a Clarus 580 GC (PerkinElmer, Waltham, MA, USA) coupled to a Clarus GC/MS SQ (PerkinElmer), in full scan mode (50 to 600 amu). The identifications of the volatile compounds were obtained matching the mass spectra with the NIST Mass Spectral Library 2.0 (NIST, Gaithersburg, MD, USA) and confirmed by calculating the retention index, as proposed by Lee et al. [22], referred to a series of *n*-hydrocarbons (C8–C40 *n*-alkanes, Sigma Aldrich), compared with those present in the NIST Chemistry WebBook (http://webbook.nist.gov/chemistry/). The semi-quantitative results were calculated by means of the Turbomass 6.1.0.1963 software (PerkinElmer).

The GC apparatus was equipped with a fused silica Rxi-5ms column (30 m × 250 μm × 0.25 μm Restek, Milan, Italy). For all the EOs, excepting *A. sativum*, the oven temperature program started from 45 °C (holding 10 min) and ramped at a rate of 2.5 °C/min to 180 °C (holding 5 min); for *A. sativum* EOs, the oven temperature program started from 50 °C (holding 1 min), ramped at a rate of 5 °C/min to 145 °C (holding 15 min), ramped at a rate of 7 °C/min to 175 °C and then ramped at a rate of 4 °C/min to 250 °C (holding 15 min); the carrier gas was helium at flow 1 mL/min; the injector temperature and the transfer line temperature were set at 250 °C. A 1% *v*/*v* solution of the EOs sample in hexane was prepared and 1 µL was injected in a splitless mode.

### 2.4. Antioxidant Capacity and Total Phenolic Content

To assess the antioxidant capacity and total phenolic content, 0.2–2 mg/mL methanolic solutions of each EO were subjected to the different spectrophotometric assays carried out by a Lambda Bio 20 ultraviolet-visible (UV/vis) spectrophotometer (PerkinElmer). The different assays conditions were presented below. 

#### 2.4.1. Trolox Equivalent Antioxidant Capacity with 2,2′-azinobis-(3-ethylbenzothiazoline-6-sulfonic acid (TEAC/ABTS) Assay

The TEAC/ABTS assay was determined as described by Masaldan et al. [23]. The TEAC/ABTS results of the samples were estimated in terms of mmol Trolox equivalent (TE)/g EO as the mean of three replicates.

#### 2.4.2. Ferric Reducing Antioxidant Power (FRAP) Assay

The FRAP was determined by using the potassium ferricyanide-ferric chloride method described by Oyaizu [24]. The FRAP of the samples was estimated in terms of mg Trolox equivalent (TE)/g EO as the mean of three replicates.

#### 2.4.3. 2,2-diphenyl-1-picrylhydrazyl (DPPH) Assay

The radical-scavenging activity of the EOs methanolic solutions was measured according to the method described by Brand-Williams et al. [25]. The DPPH results were expressed in terms of µg Trolox equivalent (TE)/g EO as the mean of three replicates.

#### 2.4.4. Total Phenolic Content (TPC)

TPC was determined by the Folin-Ciocâlteu method described by Lateef Gharib & Teixeira da Silva [26]. The TPC results were expressed in terms of mg Gallic acid equivalents (GAE)/g EO as the mean of three replicates.

### 2.5. Antimicrobial Activity

#### 2.5.1. Microbial Strains and Growth Conditions

Thirteen strains, listed in Table 1, and belonging to the Faculty of Bioscience and Technology for Food, Agriculture and Environment collection, were employed in the assessment of antimicrobial activity. The strains were stored at −80 °C in cryovials, containing anti-freezing agent (glycerol 20% *v*/*v* Sigma) and periodically confirmed by means of plate counts. Before each trial, bacterial strains were cultured overnight in Tryptone Soy agar medium (TSA, Oxoid Thermofisher, Rodano, Italy); after 24–48 h, the cells were inoculated into Tryptone Soy broth (TSB, Oxoid Thermofisher) and incubated to obtain a working fresh culture (early stationary phase). Fresh cultures were collected by centrifugation at 1300 rpm (Eppendorf-Centrifuge 5415D, Hamburg, Germany) for 5 min and washed for three times with phosphate buffer saline (50 mM pH 7.0). Inocula were standardized at about 5 × 10^5^ CFU/mL, by means of Lambda Bio 20 spectrophotometer (PerkinElmer). Strains origin and incubation conditions were also presented in Table 1.

#### 2.5.2. Determination of Minimal Inhibitory Concentration

The EOs were investigated for their Minimal Inhibitory Concentration (MIC) values according to the microdilution method, as described by Clinical and Laboratory Standards Institute (CLSI) guidelines [27]. The EOs were dissolved in PBS (Phosphate Buffer Saline) 50 mM pH 7.0 and Tween 80 (1%) to reach the initial concentration of 4.0%; working emulsions were obtained by vortexing for 5 min. The emulsions were sterilized through 0.22 µm politetrafluoroetilene (PTFE) Minisart syringe filter (Sartorius, Göttingen, Germany). The inocula were prepared as described in Section 2.5.1. A positive (100 μL of TSB medium and 100 μL inoculum) and a negative control (200 μL of sterile TSB medium) were considered for each strain. The lowest EOs concentrations that prevented growth after 48 h of incubation, was interpreted as the MIC. The Minimum Bactericidal Concentration (MBC) was determined by inoculating the content of wells were no growth was observed, on TSA plates and by incubating the plates at the temperatures reported in Table 1. The MBC was recorded as the lowest concentration not allowing bacterial growth on plates [28]. 

### 2.6. Statistical Analysis

Experimental results were expressed as means ± standard deviations. Data obtained were subjected to ANOVA (analysis of variance), and a Tukey’s HSD post-hoc test was applied at *p* < 0.05, using Microsoft Xlstat 2016 statistical software (Addinsoft, Paris, France). Correlations between TPC and AOC (antioxidant capacity) results and antimicrobial activities, were calculated using Microsoft Xlstat 2016 statistical software (Addinsoft) by means of Pearson Correlation.

## 3. Results & Discussion

### 3.1. Essential Oil Extractions

The extraction yields were calculated considering the mass (g) of the obtained EOs and the mass (g) of dried material processed. The yield results were expressed as the mean of the three replicates of the extraction ± standard deviation.

*R. officinalis*, *O. vulgare*, *S. officinalis*, *M. piperita*, *A. sativum*, *F. vulgare*, *C. sativum*, *S. montana*, *T. vulgaris* yields % were: 0.487 ± 0.011, 2.900 ± 0.012, 0.249 ± 0.001, 1.034 ± 0.057, 0.371 ± 0.011, 0.340 ± 0.016, 0.265 ± 0.010, 0.491 ± 0.027, 1.506 ± 0.096, respectively. The obtained yields were similar to ranges usually reported in the literature for the same species [26,29,30,31,32,33]. 

### 3.2. Chemical Characterization

The chemical compositions of the different EOs were reported in Table 2.

Regarding *R. officinalis* EO, the components identified were in accordance with those reported by other authors [34,35,36] for this officinal plant. The major components (*p* < 0.05) of the mixture were camphor (22%), α-pinene (17%), 1,8-cineole (16%) and borneol (12%). These approximately equal ratios have been already recognized to be characteristic of some *R. officinalis* species from Italy and nearby countries [15,37]; however, 1,8-cineole chemotype is generally reported for rosemary plants cultivated in different other locations of Italy [35,37,38].

For *O. vulgare* EO, the major constituent (*p* < 0.05) was thymol (40%), followed by carvacrol (16%), γ-terpinene (9%) and *p*-cymene (8%). The obtained data were similar with those reported by De Martino et al. [39] for thymol/carvacrol chemotypes cultivated in a southern region of Italy, by Vazirian et al. [40] and Daferera et al. [41] for Iranian and Greek oregano EOs, respectively. The main compound of *O. vulgare* EO is usually carvacrol or thymol, while other authors stated that camphor was the main constituent [15,42,43].

*S. officinalis* EO showed α-tujone (41%) as main constituent (*p* < 0.05), followed by camphor (12%) and equal ratios (*p* > 0.05) of 1,8-cineole/isocaryophyllene/humulene/ledene (total abundance of 42%). The chemical composition was in accordance with those reported in literature by different authors for some European and Iranian sages [44,45], anyhow in these works lower concentrations of α-tujone were recorded.

For *A. sativum* EO a total of six compounds were identified. The principal component (*p* < 0.05) was diallyl disulfide (65%), followed by diallyl trisulfide (20%), in accordance with the results reported by Dziri et al. [46]. These compounds represent two of the main compounds produced during the thermal or long-term decomposition of allicin, the unstable garlic main constituent released from the alliin upon an injury and by means of the activity of the enzyme alliinase [47]. 

*T. vulgaris* EO presented thymol (44%) as major compound (*p* < 0.05), thus in our case the thyme chemotype was thymol. In scientific literature there are contrasting data about the area cultivation-related chemotype, being *T. vulgaris* characterized by an evident chemotype variation that lead to different monoterpene co-occurrence and composition [48]. 

Regarding *F. vulgare* EO, the major component (*p* < 0.05) was estragole (45%), followed by similar values (*p* > 0.05) of fenchone (10%) and α-phellandrene (10%). For *M. piperita*, menthol (53%) was the EO main compound (*p* < 0.05), followed by menthofuran (8%) and menthone (7%). (S)-(+)-Linalool was the principal constituent (*p* < 0.05) of *C. sativum* EO obtained from plant seeds, for which a 77% of the total volatile mixture was accountable. In *S. montana* EO, carvacrol was the compound with the highest (*p* < 0.05) relative abundance (54%). For these last four EOs, the obtained data were in accordance with those reported in the literature from other authors [49,50,51,52,53,54].

The GC-MS chemical characterization of the analyzed samples allowed to identify the main components of the extracted EOs (total identified compounds > 90%); in addition, the data comparison with scientific literature underlined the influence of the environmental conditions of plant cultures on the composition of their volatile fraction.

### 3.3. Total Phenolic Content and Antioxidant Activity

Total phenolic content (TPC) results were showed in Table 3. The highest TPC (*p* < 0.05) was recorded for *T. vulgaris* EO (6.42 mg GAE/g EO), followed by *O. vulgare* (4.69 mg GAE/g EO) and *S. montana* (4.40 mg GAE/g EO). The other EOs showed lower values (*p* < 0.05) with a TPC range of 0.05–0.28 mg GAE/g EO and no significant differences among them (*p* > 0.05).

Antioxidant activity (FRAP, DPPH, TEAC/ABTS) results were also reported in Table 3. The different assays established that *O. vulgare*, *S. montana* and *T. vulgaris* were the EOs with the best antioxidant capacity according to free radical scavenging methods (DPPH and TEAC/ABTS). The FRAP assay underlined also the antioxidant potential of *R. officinalis* EO.

Even if the vegetal matrix had different origins, the methodology of the investigation and expression of results made difficult a direct comparison between TPC and AOC data and the literature. The presented ranges were similar to those presented by several authors for different essential oils [15,20,26,43,55].

A Pearson correlation test between the AOC and TPC data was carried out and the coefficients results obtained were presented in Table 3. As reported, among the different assays, a moderate (TPC with FRAP and ABTS) and strong (TPC with DPPH) positive correlation were revealed. Thus, the detected antioxidant activity could be attributed to total phenols content assessed in the EOs. These positive correlations between AOC and TPC have been already reported for plant species of the Mediterranean area [56].

### 3.4. Antimicrobial Activity

The antibacterial activity of the tested EOs against selected Gram-positive and Gram-negative bacteria was reported in Table 4. The strains were selected among spoiling (*P. fluorescens*, *B. thermosphacta* and *E. faecium*) and pathogenic (*Salmonella enterica* serotype Enteritidis and *Salmonella enterica* serotype Typhimurium, *L. monocytogenes* and *S. aureus*) bacteria commonly isolated from food products of different origins, to have an overview of the potentiality of the selected essential oils. 

Generally, the results observed as MIC were also confirmed as MBC. In some cases, the MIC concentration had to be doubled to obtain a bactericidal activity (i.e., MBC of *T. vulgaris* and *C. sativum* EOs was respectively 5 and 10 µL/mL for both *B. thermosphacta* B1 and B2).

For *F. vulgare* EO, the results established that the obtained EO was unable to inhibit the tested microbial strains at concentrations lower or equal than 20 µL/mL. The results were in accordance with those reported by Çetin et al. [57], who assessed that for fennel EOs obtained from the aerial parts, as in our case, the different MIC values ranged from 31.25 to 500 µg/mL. Lower MIC values could be obtained from EOs extracted from seeds [42,58] and fruits [59] of the plant; in these cases, the EOs main compound is trans-anethol, present only in minor quantities in our EO.

Regarding *R. officinalis*, *S. officinalis*, *M. piperita* and *A. sativum*, the EO tested showed a limited spectrum of activity; good results (5 μL/mL) were obtained against different strains, however higher MIC values obtained for the other strains belonging to the same species seemed to underline a strain-dependent activity. Thus, for these EOs, a variable antimicrobial activity against the Gram-negative and Gram-positive bacteria tested, was generally recognized at concentrations higher than 10 μL/mL. Nevertheless, significant results were observed for *A. sativum* EO against the two *Listeria monocytogenes* type strains. Good results were also obtained for *R. officinalis* EO; in fact, it usually shows a good antioxidant activity [60] and a lower antimicrobial activity with respect to other EOs such as oregano, thyme or tea tree [43], while in our case it showed good inhibitory activity, particularly on *L. monocytogenes* ATCC7644 and *S. aureus* STA47. This activity is probably due to the presence of camphor and 1,8-cineol among the principal constituents, as reported in Table 2.

For *O. vulgare*, the MIC values showed a wide spectrum of activity, ranging from 1.25 to 10 μL/mL for both Gram-positive and Gram-negative bacteria. In this case, the antimicrobial activity could be related to the important presence of thymol and carvacrol in the volatile fraction, as described in Section 3.1 (Table 2); the high contents of thymol, in fact, results in good antimicrobial activities [39,61]. 

The most effective EOs were those obtained from *S. montana*, *T. vulgaris* and *C. sativum.* For these EOs the MIC values ranged from 0.625 to 5 μL/mL. For these EOs thymol, (S)-(+)-linalool, and carvacrol have been already confirmed to be responsible for their antimicrobial activity, with ranges similar to those obtained in the present study [43,62,63,64]. In particular, while the antimicrobial activity of *O. vulgare* and *T. vulgaris* EOs against *L. monocytogenes* is well known [65,66], very interesting results were observed for *C sativum* against *Listeria*, but also the other tested pathogens, such as *S. aureus* (especially strain STA32) and *Salmonella* strains. These results are particularly significant, as Gram-negative bacteria are usually less sensitive to EOs than Gram-positive, because of the presence of the lipopolysaccharide layer, that provides a higher resistance to hydrophobic compounds such as essential oils [67]. On the contrary, the Gram-positive *B. thermosphacta* strains B1 and B2 were among the most resistant strains, nevertheless, they were inhibited by low concentrations of *T. vulgaris* and *S. montana* EOs.

## 4. Conclusions

The results obtained from this study established that the essential oils obtained from Abruzzo region officinal plants, mainly from *T. vulgaris*, *S. montana* and *C. sativum*, showed interesting biological potentiality. The antimicrobial and antioxidant properties assessed are excellent bases for further in vitro assays that could be used to define these essential oils as potential candidates for natural biopreservatives in combination with or in substitution to synthetic chemical ones. Moreover, additional studies should be undertaken in order to understand their potentiality in model systems and in real food samples. Study should particularly aim to establish the most effective EO concentration depending on the food matrix, its organoleptic properties, and the microorganisms it should inhibit. Anyhow, these results represent a valid basis for future evaluations and enriched current understanding about the specificity of Abruzzo region plant species and their essential oils.

## Figures and Tables

**Table 1:** Strains employed for the trial and culture and standardization conditions.

Strains		Origin	Incubation Temperature (°C)	Incubation Time (h)
*Pseudomonas fluorescens*	P34	Dairy products	28	24
*Brochothrix thermosphacta*	B2	Poultry meat	30	48
*Brochothrix thermosphacta*	B1	Poultry meat	30	48
*Salmonella* Enteritidis	S2	Meat	37	24
*Salmonella* Typhimurium	S4	Meat	37	24
*Enterococcus faecium*	P14	Fish	30	48
*Enterococcus faecium*	ATCC 19434	Type strain	30	48
*Listeria monocytogenes*	LM 4	Meat products	37	48
*Listeria monocytogenes*	ATCC 19144	Type strain	37	48
*Listeria monocytogenes*	ATCC 7644	Type strain	37	48
*Staphylococcus aureus*	STA 32	Dairy products	37	48
*Staphylococcus aureus*	STA 47	Dairy products	37	48
*Staphylococcus aureus*	STA 39	Dairy products	37	48

**Table 2:** Gas Chromatography-Mass Spectrometry (GC-MS) characterization of essential oils (EOs).

ID	RID	RIE	*R. officinalis*	*O. vulgare*	*S. officinalis*	*A. sativum*	*F. vulgare*	*M. piperita*	*C. sativum*	*S. montana*	*T. vulgaris*
Diallyl sulfide	848	847	-	-	-	0.55 ± 0.04 ^c^	-	-	-	-	-
Methyl allyl disulfide	910	911	-	-	-	0.29 ± 0.00 ^c^	-	-	-	-	-
α-Pinene	939	939	16.64 ± 0.22 ^b^	0.47 ± 0.02 ^i^	1.20 ± 0.10 ^f,g^	-	5.18 ± 0.73 ^c^	0.86 ± 0.03 ^e^	0.28 ± 0.00 ^e^	-	0.37 ± 0.01 ^f,g^
Camphene	953	956	3.39 ± 0.04 ^f^	-	1.38 ± 0.03 ^f^	-	-	-	-	-	-
Thuja-2,4(10)-diene	957	960	0.32 ± 0.01 ^l^	-	-	-	-	-	-	-	-
1-Octen-3-ol	978	979	-	0.57 ± 0.02 ^h,i^	-	-	-	-	-	1.43 ± 0.02 ^e^	-
β-Pinene	979	981	2.35 ± 0.03 ^g^	-	2.77 ± 0.16 ^e^	-	1.03 ± 0.05 ^e^	0.52 ± 0.01 ^e^	-	-	1.87 ± 0.09 ^f^
β-Myrcene	992	993	0.72 ± 0.01 ^k^	-	-	-	0.65 ± 0.06 ^e^	-	0.28 ± 0.03 ^e^	0.55 ± 0.03 ^e^	0.56 ± 0.02 ^g,h^
α-Phellandrene	1005	1006	-	-	-	-	10.49 ± 0.02 ^b^	-	-	-	-
*trans*-β-Ocimene	1015	1014	0.38 ± 0.02 ^l^	-	-	-	-	-	-	-	-
*p*-Cymene	1024	1021	1.75 ± 0.01 ^i^	8.30 ± 0.02 ^c^	0.52 ± 0.03 ^g^	-	3.33 ± 0.24 ^d^	-	0.31 ± 0.00 ^e^	10.78 ± 0.41 ^b^	18.57 ± 0.71 ^b^
Limonene	1029	1029	-	-		-	4.56 ± 0.38 ^c,d^	-	0.33 ± 0.00 ^e^	0.69 ± 0.04 ^e^	-
1,8-Cineole	1032	1034	15.71 ± 0.18 ^c^	-	10.02 ± 0.36 ^c^	-	-	5.35 ± 0.38 ^c,d^	-	0.53 ± 0.01 ^e^	-
γ-Terpinene	1060	1061	0.46 ± 0.02 ^k,l^	9.38 ± 0.01 ^c^	-	-	-	-	1.19 ± 0.07 ^d,e^	6.46 ± 0.71 ^c^	4.92 ± 0.13 ^c,d^
*cis*-Sabinene hydrate	1066	1064	-	-	-	-	-	-	-	-	3.17 ± 0.03 ^e^
Diallyl disulfide	1080	1079	-	-	-	20.16 ± 2.84 ^b^	-	-	-	-	-
Terpinolene	1087	1085	-	-	-	-	-	-	-	-	4.54 ± 0.06 ^d^
Fenchone	1088	1088	-	-	-	-	10.12 ± 0.07 ^b^	-	-	-	-
(S)-(+)-Linalool	1100	1099	2.02 ± 0.01 ^h^	1.95 ± 0.10 ^e,f^	-	-	-	0.86 ± 0.04 ^e^	77.07 ± 1.82 ^a^	2.09 ± 0.10 ^d,e^	0.37 ± 0.01 ^g,h^
α-Thujone	1102	1104			30.46 ± 0.49 ^a^						
1-Octen-3-ol, acetate	1110	1113	-	-	-	-	-	-	-	-	1.27 ± 0.01 ^f,g^
Menthone	1126	1116	-	-	-	-	-	6.87 ± 0.11 ^b,c^	-	-	-
l-Pinocarveol	1135	1132	0.23 ± 0.01 ^l^	-	-	-	-	-	-	-	-
Camphor	1139	1137	22.07 ± 0.23 ^a^	-	11.53 ± 0.34 ^b^	-	-	-	2.60 ± 0.10 ^c,d^	-	0.29 ± 0.02 ^h^
Borneol	1162	1160	11.99 ± 0.04 ^d^	0.66 ± 0.01 ^g,h,i^	3.92 ± 0.02 ^d^	-	-	-	0.48 ± 0.01 ^e^	4.51 ± 0.37 ^c,d^	-
Menthofuran	1164	1165	-	-	-	-	-	7.81 ± 0.59 ^b^	-	-	-
Menthol	1171	1178	-	-	-	-	-	53.39 ± 0.24 ^a^	-	-	-
Isocamphopinone	1175	1174	1.08 ± 0.00 ^j^	-	-	-	-	-	-	-	-
Terpinene-4-ol	1179	1176	0.26 ± 0.01 ^l^	0.38 ± 0.01 ^i^	1.43 ± 0.09 ^f^	-	-	-	0.55 ± 0.03 ^e^	-	-
2-Vinyl-1.3-dithiane	1182	1084	-	-	-	4.60 ± 0.09 ^c^	-	-	-	-	-
Isomenthol	1194	1192	-	-	-	-	-	-	-	-	-
α-Terpineol	1195	1195	1.49 ± 0.08 ^i^	1.64 ± 0.04 ^f,g,h,i^	0.51 ± 0.01 ^g^	-	0.49 ± 0.03 ^e^	-	0.59 ± 0.03 ^e^	1.61 ± 0.02 ^e^	4.48 ± 0.09 ^d^
Myrtenol	1196	1194	2.35 ± 0.03 ^g^	-	-	-	-	-	-	-	2.82 ± 0.15 ^e^
Estragole	1199	1198	-	-	-	-	44.86 ± 0.26 ^a^		-	-	-
Verbenone	1205	1203	0.44 ± 0.05 ^l^	-	-	-	-	-	-	-	-
Isopulegone	1237	1237	-	-	-	-	-	2.01 ± 0.03 ^e^	-	-	-
Piperitone	1253	1250	-	-	-	-	-	0.21 ± 0.02 ^e^	-	-	-
*cis*-Geraniol	1254	1256	-	-	-	-	-	-	5.24 ± 0.39 ^b^	-	-
Thymol methyl ether	1255	1257	-	1.04 ± 0.01 ^f,g,h^	-	-	-	-	-	-	-
Neomenthyl acetate	1273	1270	-	-	-	-	-	0.81 ± 0.06 ^e^	-	-	-
*trans*-anethol	1285	1282		-	-	-	6.55 ± 0.06 ^c^	-	-	-	-
Bornyl acetate	1286	1286	5.62 ± 0.01 ^e^	-	-	-	-	-	-	-	-
Thymol	1290	1290	-	40.32 ± 1.12 ^a^	-	-	-	-	-	2.53 ± 0.14 ^d,e^	43.68 ± 0.54 ^a^
Menthyl acetate	1295	1294	-	-	-	-	-	4.61 ± 0.01 ^d^	-	-	-
Carvacrol	1299	1299	-	16.20 ± 0.05 ^b^	-	-	0.63 ± 0.01 ^e^	-	1.03 ± 0.03 ^d,e^	54.17 ± 2.33 ^a^	5.51 ± 0.12 ^c^
Diallyl trisulfide	1301	1300	-	-	-	65.39 ± 2.50 ^a^	-	-	-	-	-
Isocaryophyllene	1438	1434	3.37 ± 0.20 ^f^	2.90 ± 0.07 ^e^	10.49 ± 0.00 ^c^	-	1.05 ± 0.05 ^e^	1.67 ± 0.03 ^e^	0.62 ± 0.02 ^e^	2.91 ± 0.11 ^d,e^	1.61 ± 0.03 ^f^
Humulene	1467	1467	0.72 ± 0.02 ^k^	0.77 ± 0.03 ^g,h,i^	10.01 ± 0.35 ^c^	-	0.49 ± 0.01 ^e^	-	-	-	-
Germacrene-d	1487	1490	-	0.90 ± 0.01 ^f,g,h,i^	-	-	-	0.87 ± 0.02 ^e^	-	-	0.55 ± 0.01 ^g,h^
β-Bisabolene	1506	1509	-	4.64 ± 0.06 ^d^	-	-	-	-	0.83 ± 0.04 ^d,e^	1.82 ± 0.03 ^e^	1.64 ± 0.01 ^f^
γ-Cadinene	1513	1514	-	-	-	-	-	-	-	0.54 ± 0.02 ^e^	-
δ-Cadinene	1523	1522	-	0.65 ± 0.01 ^g,h,i^	0.45 ± 0.01 ^g^	-	-	-	-	0.54 ± 0.02 ^e^	-
Diallyl tetrasulfide	1555	1557	-	-	-	1.49 ± 0.07 ^c^	-	-	-	-	-
Caryophyllene oxide	1581	1583	-	1.73 ± 0.01 ^f,g^	1.92 ± 0.05 ^e,f^	-	-	1.21 ± 0.04 ^e^	3.16 ± 0.17 ^c^	1.41 ± 0.05 ^e^	-
Ledene	1585	1589	-	-	10.12 ± 0.44 ^c^	-	-	-	-	-	-
Total identified compounds			93.35 ± 0.43	92.52 ± 0.84	97.04 ± 0.46	92.47 ± 0.39	90.44 ± 0.87	93.17 ± 0.39	94.55 ± 1.69	92.56 ± 3.94	96.22 ± 1.47

Results were expressed as mean relative abundances % of three replicates. In the table: ID, component name; RID, retention index retrieved from http://webbook.nist.gov/chemistry/ for the same analysis conditions; RIE, experimental retention index referred to C8–C40 *n*-alkane mixture standard. Statistical groups were defined by progressive alphabetical letters (case-letter). For the same matrix (same column), results followed by the same case-letter are not significantly different according to Tukey’ HSD post hoc test (*p* > 0.05).

**Table 3:** Total phenolic content (TPC) and antioxidant activity results Ferric Reducing Antioxidant Power (FRAP), 2,2-diphenyl-1-picrylhydrazyl (DPPH) and Trolox Equivalent Antioxidant Capacity with 2,2′-azinobis-(3-ethylbenzothiazoline-6-sulfonic acid (TEAC/ABTS) of EOs and Pearson correlation coefficients between the different antioxidant activity assays and total phenolic content.

Assay	TPC	FRAP		DPPH		ABTS
*Rosmarinus officinalis*	0.111 ± 0.002 ^c^	188.270 ± 0.437 ^a^		10.288 ± 0.258 ^c,d^		0.084 ± 0.001 ^e^
*Origanum vulgare*	4.688 ± 0.304 ^b^	168.220 ± 1.837 ^b^		23.963 ± 2.435 ^b^		1.765 ± 0.005 ^b^
*Salvia officinalis*	0.178 ± 0.008 ^c^	12.304 ± 0.022 ^f^		8.709 ± 0.885 ^c,d^		0.098 ± 0.005 ^e^
*Mentha piperita*	0.338 ± 0.018 ^c^	0.543 ± 0.044 ^h^		11.289 ± 0.514 ^c^		0.154 ± 0.006 ^d^
*Allium sativum*	0.050 ± 0.001 ^c^	3.924 ± 0.142 ^g^		7.868 ± 0.158 ^d^		0.037 ± 0.003 ^g^
*Foeniculum vulgare*	0.283 ± 0.013 ^c^	15.202 ± 0.175 ^e^		11.466 ± 0.636 ^c^		0.043 ± 0.003 ^g^
*Coriandrum sativum*	0.046 ± 0.004 ^c^	4.122 ± 0.241 ^g^		10.656 ± 1.043 ^c,d^		0.067 ± 0.004 ^f^
*Satureja montana*	4.398 ± 0.252 ^b^	159.280 ± 1.575 ^c^		27.015 ± 0.959 ^a^		1.997 ± 0.003 ^a^
*Thymus vulgaris*	6.419 ± 0.219 ^a^	126.869 ± 0.175 ^d^		21.751 ± 0.862 ^b^		1.131 ± 0.012 ^c^
**Pearson Correlation Coefficients**						
**Assay**	**FRAP**		**ABTS**		**DPPH**	
TPC	0.642		0.691		0.905	

Regarding TPC and AOC (antioxidant activity): results were expressed as mg Gallic acid equivalents (GAE)/g EO for TPC assay; mg Trolox equivalent (TE)/g EO for FRAP assay; µg Trolox equivalent (TE)/g EO for DPPH assay; mmol Trolox equivalent/g EO for ABTS assay. The showed values were the mean of three replicates. For the same assay (same column), results followed by the same case-letter are not significantly different according to Tukey’ HSD post hoc test (*p* > 0.05). For Pearson Correlation Coefficients: the positive/negative strength of correlation was considered: low for ±0.1 < *r* < ±0.3, moderate for ±0.3 < *r* < ±0.7, and strong for *r* > ±0.7; for values of *r* < ±0.1 the variables were considered not correlated.

**Table 4:** Minimum Inhibitory Concentration (MIC) (μL/mL) of selected essential oils against the different strains.

Strains		*Rosmarinus officinalis*	*Origanum vulgare*	*Salvia officinalis*	*Mentha piperita*	*Allium sativum*	*Foeniculum vulgare*	*Satureja montana*	*Thymus vulgaris*	*Coriandrum sativum*
*P. fluorescens*	P34	10	1.25	10	>20	10	>20	1.25	2.5	5
*B. thermosphacta*	B2	>20	10	>20	>20	>20	>20	2.5	2.5	5
*B. thermosphacta*	B1	>20	10	>20	>20	>20	>20	5	2.5	5
*S.* Enteritidis	S2	20	2.5	>20	>20	>20	>20	1.25	1.25	5
*S.* Typhimurium	S4	>20	2.5	>20	>20	>20	>20	5	2.5	5
*E. faecium*	P14	>20	5	>20	>20	>20	>20	5	5	5
*E. faecium*	ATCC 19434	10	5	>20	>20	10	>20	5	2.5	2.5
*L. monocytogenes*	LM 4	>20	5	10	>20	>20	>20	2.5	1.25	0.625
*L. monocytogenes*	ATCC 19144	10	5	5	20	2.5	>20	2.5	1.25	0.625
*L. monocytogenes*	ATCC 7644	5	10	10	10	2.5	>20	5	1.25	0.625
*S. aureus*	STA 32	10	5	>20	>20	10	>20	2.5	2.5	0.625
*S. aureus*	STA 47	5	5	10	2.5	5	>20	5	1.25	1.25
*S. aureus*	STA 39	>20	5	>20	>20	10	>20	5	1.25	1.25
